# Experimental Studies on the Differentiation of Fibroblasts into Myoblasts induced by MyoD Genes *in vitro*

**Published:** 2008-03

**Authors:** Zhongmin Liu, Huimin Fan, Yang Li, Song Guo Zheng

**Affiliations:** 1 *Division of Cardiology Surgery, Shanghai East Hospital, Tongji University School of Medicine, Shanghai, P. R. China;*; 2 *Department of Medicine, Keck School of Medicine, University of Southern California, Los Angeles, USA*

**Keywords:** lentivirus, fibroblast, myogenesis, MyoD, Cx43

## Abstract

To evaluate the biological functions of myogenic regulatory factors, we have examined the effects of ectopic expression of MyoD and Cx43 genes in the fibroblasts on the differentiation of myoblast *in vitro*. The expression of MyoD and Cx43 in the transfectants was confirmed by RT-PCR and Western blot. More than 50% of fibroblasts transfected with MyoD or both MyoD and Cx43 genes displayed typical morphological features of myoblast-like cells at 20 days following gene transfection, including cell elongation, cytoplasm enrichment and granule manifold. Moreover, these myoblast-like cells also expressed both desmin and α-actin. These results demonstrate that direct exogenous expression of the myogenic regulatory factors is sufficient to induce transdifferentiation of fibroblasts into a myoblast-like lineage and provide new insights into the trauma repair after myocardial infraction.

## INTRODUCTION

Heart failure caused by myocardial infarction is one of the key causes of death in the world (1). Cardiomyocytes are final differentiated cells that lose the ability to regenerate. After myocardial infarction, injured cardiomyocytes are replaced by fibrotic tissue promoting the development of heart failure (2). Although drug therapy and heart transplantation surgery provide the main clinical options, the effect of these approaches is unsatisfactory so far.

Cell transplantation has been emerging as an innovative therapeutic approach to treat acute myocardial infarction and heart failure (3-5). This process has led us to consider cell therapy as a new treatment option for myocardial tissue regeneration after ischemic damage. Autologous skeletal myoblasts (or satellite cells) and bone marrow myogenic stem cells are committed to optimal progenitor cells (6). They are capable of regenerating and incorporating into cardiomyocytes and enabling heart to restore the normal function (7). However, tiny number of autologous satellite or bone marrow stem cells has been proved problematic in using these cells as progenitor cells. It is desirable to develop an alternative approach for this cell transplantation therapy.

The activity of myogenic regulatory factors (MRFs), such as MyoD and myogenin also determines myogenesis and reduction of activity of MRFs also suppress myogenesis (8, 9). MyoD determines myoblast cell fate, and myogenin acts downstream of MyoD by regulating commitment to myogenic differentiation (10). Connexin 43 (Cx43), the main protein in the gap junction of cardiomyocytes, is closely related to the intercellular GJIC (gap junctional intercellular communication) which is necessary for the synchronous contraction of transplanted cells and cardiomyocytes (11).

It has been reported that the ectopic expression of MyoD gene in non-muscle cells can activate endogenous gene expression and thus promote the muscle formation (12). Fibroblasts are the most abundant cell type in normal connective tissues located under the basal lamina of adult skeletal muscle and have the ability to transdifferentiate into myofibroblast (13, 14). In the current study, we try to assess the effect of the lentivirus delivery of MyoD and Cx43 genes on the differentiation of fibroblasts into myoblast-like cells. Interestingly, the stable expression of these genes in the transferred fibroblasts enables cells to transdifferentiate into myoblast-like cell phenotypes. Thus, MyoD and Cx43 genes may play an important role in the cellular transplant cardiomyoplasty. As fibroblasts are easy to be isolated from patients and can be transferred into myoblast without leading to major ethical and technical problems, they have great potential as therapeutic agents.

## MATERIALS AND METHODS

### Reagents

High glucose DMEM (Dulbecco’s Modification of Eagle’s Medium) and fetal bovine serum were purchased from GIBCO (USA). Virapower Lentiviral Expression System kit was from Invitrogen (USA). Restriction enzymes BamH1, Xho1 and T4 ligase were purchased from New England Biolabs, Inc. (USA). Plasmid XL1-blue was kindly provided by Dr. Zhao (Shanghai, China). RT-PCR reagent kit was purchased from Promega (USA). Rabbit primary polyclonal antibody against MyoD and Cx43 were obtained from Santa Cruz (USA), while rabbit anti-rat Desmin and α-actin were from Lab Vision. PE-conjugated goat secondary antibody IgG was purchased from Sigma Chemical (St. Louis, MO).

### Cell culture

Rat fibroblast cell line RFL-6 was provided by institute of Biochemistry and Cell biology, Chinese Academy of Sciences. The cells were routinely cultured in Dulbecco’s Modification of Eagle’s Medium containing 10% fetal calf serum (FCS) at 37°C in a humidified atmosphere of 5% CO2 and 95% air. The cells were seeded into T-25 cm^2^ or T-75 cm^2^ in DMEM containing 10% FCS. To obtain the desired quantity of RFL-6 cells, 0.25% trypsin and 0.02% EDTA were added to cultures. Experimental groups consist of several ones including non-transfected cells, Cx43-transfected cells, MyoD-transfected cells and MyoD-and-Cx43-transfected cells.

### Vector construction and lentivirus transfection

Rat Cx43 and MyoD total gene sequences were synthesized by Shanghai Yinjun biotechnological co. and cloned into plasmid p18-T and transformed into *E. coli* DH5α strain. The plasmids were cut by restriction enzymes BamH1, Xho1 and target gene sequences were then cloned into vector pENTR11. The entry clone was confirmed by sequencing. The entry clone (300 ng) and pLenti-DEST vector (300 ng) were dissolved by LR clonase reaction buffer 4ul and TE buffer 12 ul; 4ul LR clonase enzyme was added to the sample above and incubated at 25°C for 1 hour, then 2 ul proteinase K solution was added to the reaction and incubated for 10 minutes at 37°C, which yielded expression clone. The expression clone was transformed into Stbl3 *E. coli* strain, selected in LB agrose plates containing 100 ug/ml ampicillin and 50ug/ml Blasticidin. The gene of interest was further confirmed using sequencing with primers: 5’-CGCAAATGGGCGGTAGGCGTG-3’ (forward), 5’-ACCGAGGAGAGGGTTA GGGAT-3’ (reverse). The pLenti-based expression vector containing gene of interest and the ViraPower packing mix were co-transfected into the 293FT cell line to produce a lentiviral stock by demonstrating the Invitrogen Lentiviral expression system kit procedure. On the day of transduction, lentiviral stock was thawed and the appropriated amount of virus was diluted and added into fresh complete medium, the culture medium from the cells was harvested. The medium containing virus was mixed gently and added to the cells and incubated at 37°C overnight. The following day, the culture medium was replaced with fresh, complete medium containing 50 ug/ml Blasticidin to select stably transduced cells until Blasticidin-resistant colonies were identified. Five colonies were picked up and expanded, and the expression of the recombination protein was determined using Western blot.

### RT-PCR

Expression of rat Cx43 and MyoD mRNA was analyzed by the RT-PCR method as previously described (21). Primers for Cx43 were 5’-TCT GTG CCC ACC CTC CTG TA-3’ (sense), 5’-AGA GTG GAG CCG TTG GTG AG-3’ (antisense), while primers for MyoD were 5’-TGG CAG TGA GCA CTA CAG C-3’(sense), 5’-CAT CTG GCA AAA GCA GCG AAG-3’(antisense). The annealing temperatures were 55°C for Cx43 and MyoD. The numbers of cycles of PCR was 35 for MyoD and myogenin. GAPDH specific primers were used as internal controls. By using human cDNA as a template, we have confirmed that the primers described as above for Cx43 and MyoD yield the expected single band (580 bp and 254 bp), respectively. Reaction products were resolved by electrophoresis through 2% agarose gels and were visualized with a laser image analyzer.

### Western Blot Analysis

The cells were washed twice with phosphate-buffered saline (PBS) and then lysed and homogenized in a lysis buffer containing 62.5 mM Tris-HCl, pH6.8, 2% sodium dodecyl sulfate (SDS), 50 mM dithiothreitol, and 10% glycerol. The cytosolic fraction was collected as the supernatant after centrifugation at 13,000 g for 60 min at 4°C. SDS–PAGE was performed in 10% polyacrylamide gel. Western blotting analysis was performed by using appropriated antibody, with peroxidase-labeled goat against mouse IgG being used as second antibody. Peroxidase activity on the PVDF membrane was visualized on X-ray film of the ECL Western blotting detection system (Pierce).

### Immunofluorescence analysis

Cells were fixed with acetone and incubated with appropriated rabbit anti-human IgG in PBS at 37°C for 1 h, washed briefly in PBS, incubated with the PE-conjugated second antibody (1:1000) and DAPI (1:200) at 37°C for 1 h. The cells were then washed in PBS and coverslips were mounted with glycerine. Samples were analyzed by fluorescence microscopy.

## RESULTS

### Culture of packaging cells 293FT and production of lentivirus

**Figure 1:**
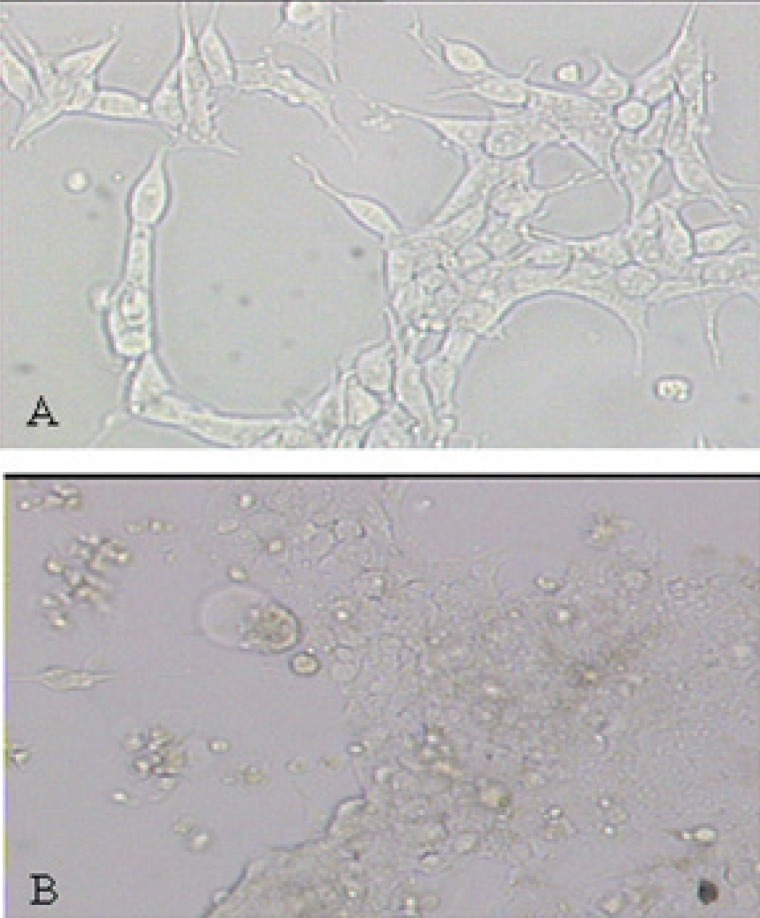
Culture of packaging cells 293FT and production of lentivirus. (A) Morphology of 293FT cells before viral packaging. The packaging cells were shown in good condition; (B) Morphology of 293FT cells during ingathering. The pLenti-based expression vector containing gene of rat Cx43 or MyoD and the ViraPower packing were transfected into 293FT cell line to produce lentivirus. The viruses were ingathered after culturing for 72h.

Constructed pLenti6/V5-DEST-Cx43 and pLenti6/V5-DEST-MyoD expressing clones were first confirmed by sequencing. 293FT cells were observed in an ideal condition when they grew to a confluence of 30-50% (Fig. 1A). Viral packaging was carried out according to Lentiviral expression system protocol. Viruses were gathered after 72 hours (Fig. 1B). The dripping degrees were 3.5 × 10^5^ cfu/ml and 2.0 × 10^5^ cfu/ml respectively.

### Stable expression of MyoD and Cx43 genes following the lentiviral transfection

**Figure 2:**
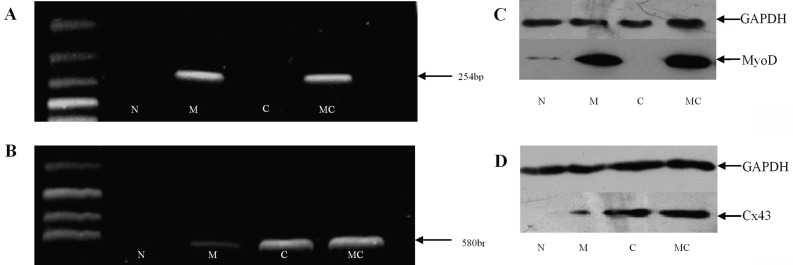
Expression of rat Cx43 and MyoD after lentivirus transfection. MyoD and Cx43 expression levels in four groups of fibroblasts: non-treated fibroblast (N), MyoD gene-transfected fibroblast (M), Cx43 gene-transfected fibroblast (C) and MyoD-Cx43-transfected fibroblast (MC) were analyzed. (A) MyoD mRNA expression was determined by RT-PCR. The 254bp band is MyoD gene amplifying product. The figure shows that MyoD mRNA was highly expressed in the MyoD- and MyoD plus Cx43- transfected fibroblasts; (B) Cx43 mRNA expression was determined by RT-PCR. The 580 bp band indicates Cx43 gene PCR product. Panel B reveals that Cx43 mRNA was only detected highly in the Cx43- transfected fibroblasts. In addition, low level expression of Cx43 was found in MyoD gene-transfected group. Non-treated fibroblasts did not express mRNA of these two genes; (C) MyoD and (D) Cx43 protein level was analyzed by western blotting. GAPDH was used as a sample loading control. MyoD or Cx43 protein was expressed in the MyoD- or Cx43-transfected fibroblasts. Note low level expression of Cx43 was found in MyoD gene-transfected fibroblasts.

MyoD or Cx43 mRNA was highly expressed on the MyoD- or Cx43-transfected fibroblasts. As shown in Fig. 2A and 2B, MyoD or Cx43 mRNA was detected by RT-PCR on the transfected fibroblasts. Conversely, non-treated fibroblasts did not express mRNA of these two genes. In addition, low levels of expression of Cx43 mRNA were found in MyoD gene-transfected group. Moreover, the levels of protein expression for MyoD or Cx43 were consistent with mRNA levels in the transfected fibroblasts by Western blot (Fig. 2C, 2D). The expression of MyoD or Cx43 proteins was further confirmed by immunocytochemistry (data not shown). The results suggest that MyoD- or Cx43-transfected fibroblasts not only expressed the mRNA but also could translate the corresponding functional protein.

### Transfected-MyoD and Cx43 genes enable fibroblast to differentiate into myoblast *in vitro*

**Figure 3:**
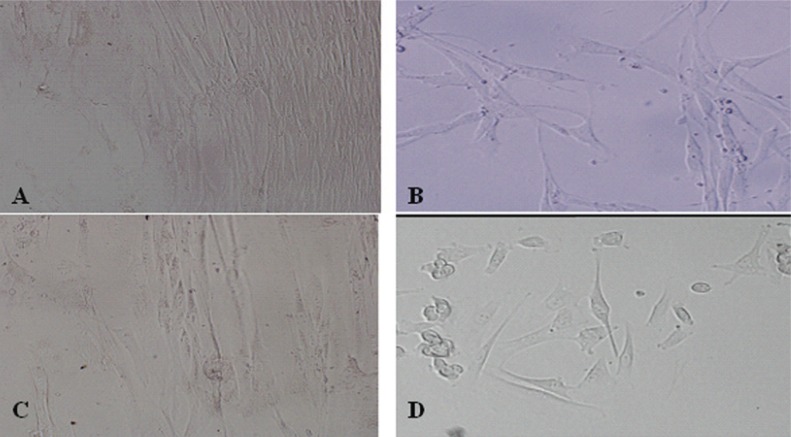
Transfected-Myo D and Cx43 genes enable morphology conversion of fibroblast to myoblast like cells. Positive clones of four groups of fibroblasts: (A) non-treated fibroblast (N), (B) MyoD gene-transfected fibroblast (M), (C) Cx43 gene-transfected fibroblast (C) and (D) MyoD-Cx43-transfected fibroblast (MC) were screened out after lentivirus with Cx43 or MyoD gene transfection into rat fibroblast cell line RFL-6. The figures show that RFL-6 cells in M and MC group had changed with cell elongation, cytoplasm enrichment and granule manifold and become myoblast like cells. Cell morphology of C group was similar with N group but proliferation ability decreased obviously (data not shown).

We also determined whether forced expression of MyoD and/or Cx43 in fibroblasts could convert these cells toward a myoblast-like cell phenotype. Lentivirus vectors expressing MyoD or Cx43 were generated (Fig. 1), and fibroblasts were treated with MyoD gene alone, Cx43 gene alone, MyoD plus Cx43 genes, or vehicle control. The cell morphology was observed daily and traced until 20 days following gene transfer. Interestingly, more than 50% of the cells transfected with MyoD or MyoD plus Cx43 have converted to myoblast-like cells, as seen by cell elongation, cytoplasm enrichment and granule manifold (Fig. 3A, B, D). However, the morphology of cells transfected with Cx43 alone was similar to control groups but proliferative ability was decreased markedly (Fig. 3C).

**Figure 4:**
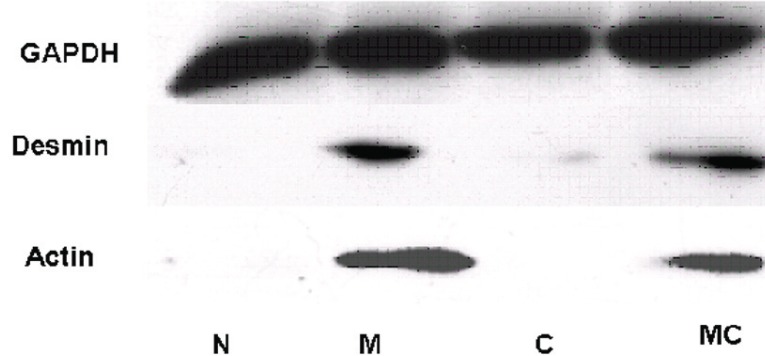
Expression of Desmin and α-actin in different transfected group were analyzed by western blotting. The antibodies of α-actin and Desmin were muscle cell specific. GAPDH was also detected as a sample loading control. The figure shows that MyoD downstream molecules, Desmin and α-actin, were all detected in cells transfected with MyoD or MyoD plus Cx43 genes but not in the cells transfected with Cx43 gene alone or control.

The development of myogenesis by MyoD gene transfer was further evaluated by following the expression of downstream molecules Desmin and α-actin, a late marker of myoblast differentiation. As expected, expression of Desmin and α-actin was detected in cells transfected with MyoD or MyoD plus Cx43 genes but not in the cells transfected with Cx43 gene alone or control vehicle (Fig. 4).

## DISCUSSION

Cellular loss and scar formation due to myocardial infarction are the main factors influencing heart function and they are the pathological basis of refractory heart failure and even death. Drug therapy, intervention therapy and surgery can ameliorate myocardial ischemia and the symptoms of heart failure, but these approaches are unable to regenerate infracted cardiomyocytes. It is desirable to find a new way to substitute the infracted cardiomyocytes and improve the heart function. Recently, with the rapid development of cellular biological engineering technique, it is becoming likely to use cell transplantation to improve heart function (15, 16). Given both cardiac muscle and skeletal muscle have similar contraction function and structure, cell transplantation might help the heart to regain or restore its function in heart failure or infarction. We propose that the transfection of MyoD into human fibroblast can induce fibroblast to become functional myocytes which can provide a novel approach to heart failure therapy.

Fibroblasts were adopted for cell transplantation due to their rich source and the ability to differentiate into myoblasts (13, 14). Thus, adequate available differentiated fibroblasts cells could be easily obtained, allowing us to overcome one of the major obstacles, namely, lack of the appropriate cell progenitors in cell transplantation. Moreover, those autologous differentiated fibroblasts will not induce transplant rejection when used for transplantation. MyoD is one of the key genes regulating muscle formation in the development of embryo (17). The role of MyoD in muscle formation lies in that its expression is a switch of other muscle formation related genes and is indispensable for the maintenance of muscle differentiation (18). Some studies have indicated that muscle formation could be started when the MyoD gene is transfected into non-muscle cells, in the mean time, expression of foreign MyoD gene could activate endogenous genes and promote muscle formation (19-21).

Our research demonstrates that fibroblasts expressing stably MyoD show morphology and expression of down-stream muscle specific molecules, Desmin and α-actin, similar to myoblasts. It is likely that we have successfully induced rat fibroblasts to differentiate into myoblasts using a Lentiviral expression system. However, it is also important to enhance the functional integration of transplanted cells and host cardiomyocytes which is still another critical problem in cellular transplant cardiomyoplasty. According to previous reports, the functional integration is dependent on the intercellular GJIC (gap junctional intercellular communication) formation between transplanted cells and host cardiomyocytes, which is necessary for the synchronous contraction (22). Connexin 43, one of the most important proteins in the gap junction, has been proved to be closely related to GJIC formation among myocardial cells (23). The research of Suzuki *et al*. indicated that the Cx43-overexpressing skeletal myoblast cell lines resulted in improved formation of functional intercellular gap junctions. They also found Cx43 could promote differentiation of myoblast (24). However, our research shows that Cx43 introduction alone cannot induce fibroblasts to differentiate into myoblasts. Cx43 does not affect the functional effect of MyoD on myoblast differentiation. It is noted that Cx43 might affect the proliferative capacity of the transfected cells due to its role as the functional regulator of GJIC. However, Cx43 could also regulate the proliferation of cells in a GJIC independent way (25). In addition, we have found traceable expression of Cx43 in MyoD transfected myoblasts; however, whether Cx43 expression is induced directly by MyoD or through another pathway remains to be investigated.

We demonstrate that the transfection of the MyoD and Cx43 genes to fibroblasts promotes the differentiation of fibroblasts into myoblasts. The expression of Cx43 in differentiated myoblasts may facilitate the formation of GJIC between myoblasts and host cardiomyocytes, which is required for the heart to contract synchronously. Thus, our research might provide a novel approach for the treatment of patients with heart failure after myocardial infarction.
